# Interoception in insula subregions as a possible state marker for depression—an exploratory fMRI study investigating healthy, depressed and remitted participants

**DOI:** 10.3389/fnbeh.2015.00082

**Published:** 2015-04-10

**Authors:** Christine Wiebking, Moritz de Greck, Niall W. Duncan, Claus Tempelmann, Malek Bajbouj, Georg Northoff

**Affiliations:** ^1^Cluster of Excellence in Cognitive Sciences, Department of Sociology of Physical Activity and Health, University of PotsdamPotsdam, Germany; ^2^Mind, Brain Imaging and Neuroethics, Institute of Mental Health Research, University of OttawaOttawa, ON, Canada; ^3^Department of Psychosomatic Medicine and Psychotherapy, University Medicine MainzMainz, Germany; ^4^Centre for Cognition and Brain Disorders, Hangzhou Normal UniversityHangzhou, China; ^5^Graduate Institute of Humanities in Medicine, Taipei Medical UniversityTaipei, Taiwan; ^6^Brain and Consciousness Research Center, Taipei Medical University-Shuang Ho HospitalNew Taipei City, Taiwan; ^7^Department of Neurology, Otto-von-Guericke University MagdeburgMagdeburg, Germany; ^8^Cluster of Excellence “Languages of Emotion” and Dahlem Institute for Neuroimaging of Emotion, Freie Universität BerlinBerlin, Germany; ^9^Department of Psychiatry, Charité-Universitätsmedizin, Campus Benjamin FranklinBerlin, Germany; ^10^Department of Psychology, National Chengchi UniversityTaipei, Taiwan

**Keywords:** major depressive disorder, interoceptive awareness, insula, remission, neuroimaging, fMRI, hopelessness, interoception

## Abstract

**Background:** Interoceptive awareness (iA), the awareness of stimuli originating inside the body, plays an important role in human emotions and psychopathology. The insula is particularly involved in neural processes underlying iA. However, iA-related neural activity in the insula during the acute state of major depressive disorder (MDD) and in remission from depression has not been explored.

**Methods:** A well-established fMRI paradigm for studying (iA; heartbeat counting) and exteroceptive awareness (eA; tone counting) was used. Study participants formed three independent groups: patients suffering from MDD, patients in remission from MDD or healthy controls. Task-induced neural activity in three functional subdivisions of the insula was compared between these groups.

**Results:** Depressed participants showed neural hypo-responses during iA in anterior insula regions, as compared to both healthy and remitted participants. The right dorsal anterior insula showed the strongest response to iA across all participant groups. In depressed participants there was no differentiation between different stimuli types in this region (i.e., between iA, eA and noTask). Healthy and remitted participants in contrast showed clear activity differences.

**Conclusions:** This is the first study comparing iA and eA-related activity in the insula in depressed participants to that in healthy and remitted individuals. The preliminary results suggest that these groups differ in there being hypo-responses across insula regions in the depressed participants, whilst non-psychiatric participants and patients in remission from MDD show the same neural activity during iA in insula subregions implying a possible state marker for MDD. The lack of activity differences between different stimulus types in the depressed group may account for their symptoms of altered external and internal focus.

## Introduction

The insula has been described as a brain region serving as an interface between external stimuli and stimuli originating inside the body (Craig, 2002, 2009, 2011; Farb et al., 2013; Simmons et al., 2013). Under one model, a gradual integration of intero-/exteroceptive stimuli occurs along a pathway running from the posterior to the anterior parts of the insula (Craig, 2009). Such a model posits that the insula as a whole is formed from separate but highly interconnected modules. This is in line with the major cytoarchitectonic structure of the insula (Mesulam and Mufson, 1985; Morel et al., 2013). It is also supported by recent imaging studies that have used task-based functional imaging (Deen et al., 2011; Chang et al., 2013), resting-state functional connectivity (Sridharan et al., 2008; Touroutoglou et al., 2012) and diffusion tractography structural connectivity (Cerliani et al., 2012; Cloutman et al., 2012) to parcellate the insula into a threefold regional organization (Deen et al., 2011; Chang et al., 2013). It remains to be shown, however, whether these subregions show differential neural responses to interoceptive awareness (iA).

In line with former research and prominent theories of emotional processing, the insula has been suggested to play a key role in the connection between iA and affective experience (Damasio, 1999; Craig, 2002; Bechara and Naqvi, 2004; Critchley et al., 2004; Wiens, 2005; Lamm and Singer, 2010; Paulus and Stein, 2010). Using functional magnetic resonance imaging (fMRI), this linkage was recently directly demonstrated in healthy participants, where neural activity during an iA task and emotional processing was found to overlap within the insula (Zaki et al., 2012). Such a connection between iA-related activity in the insula and emotional processing is suggestive of the insula being involved in mood disorders such as major depressive disorder (MDD). This disorder is characterized by extreme negative affect, somatic symptoms, altered body awareness (Henningsen et al., 2003; Nyboe Jacobsen et al., 2006) and feelings of hopelessness (Bjärehed et al., 2010). The assumption that both somatic signals and interoception are altered in depression is further supported by a recent literature review on “Interoceptive Dysfunction: toward an Integrated Framework for Understanding Somatic and Affective Disturbance in Depression,” suggesting that depressed symptoms may arise from a disturbed integration of intero-/extero-ceptive stimuli (Harshaw, 2015). Though the insula mirrors a brain region constantly found in neuroimaging findings regards depression (Fitzgerald et al., 2008), few studies have applied an iA-related task in depressed populations yet. For example, Avery reported reduced neural activity during iA in MDD, whilst this reduced activity was inversely associated with severity of depression and somatic symptoms (Avery et al., 2014). Similarly, own studies showed altered body perception and aberrant insula activity in MDD (Wiebking et al., 2010) with negative associations between iA and hopelessness in the insula in non-psychiatric participants (Wiebking et al., 2014b), which are all factors typically affected in depression (Bjärehed et al., 2010; Paulus and Stein, 2010). As well as such links between insula activity, behavioral and subjective aspects of depression, changes in insula structure (Sprengelmeyer et al., 2011), metabolism (Brooks et al., 2009) and regional homogeneity (Yao et al., 2009; Liu et al., 2010; Guo et al., 2011), as compared to healthy participants, have all been reported in depression (see Sliz and Hayley, 2012 for a review).

Taking these findings together, it can be hypothesized that the insula plays a crucial role in integrating emotional and interoceptive stimuli and that these processes are disrupted in mental disorders (Paulus and Stein, 2010). Studies targeting the insula during iA in depressed individuals are rare (Wiebking et al., 2010; Avery et al., 2014) and support the hypothesis of aberrant neural activity during interoceptive processing in the insula. However, studies exploring whether any iA-related changes in MDD return to activity levels seen in healthy groups when in remission from depression remain to be investigated. As such, in the present study we aimed to compare neural responses during iA in functional subregions of the insula between independent groups of healthy, depressed and remitted individuals. Given that feelings of hopelessness are a major symptom of MDD, we sought to investigate the relationship between insula activity and hopelessness.

To these ends, a well-established intero-/exteroceptive awareness task was used in fMRI (Critchley et al., 2004; Pollatos et al., 2007; Wiebking et al., 2014a,b). The insula was divided into three subregions, as defined previously through functional connectivity pattern clustering (Deen et al., 2011; also used by Uddin et al., 2013). Exteroceptive awareness (eA; tone counting) and undirected awareness (noTask) were used as control conditions for activity comparisons and for correlations with subjective hopelessness scores. It was hypothesized that participants after remission from depression and non-psychiatric controls show comparable BOLD responses during iA in the insula, whilst depressed participants would show decreased BOLD responses with a reduced differentiation between the different task conditions (iA, eA, noTask).

## Methods

### Participants and psychometrics

Using fMRI, three independent study groups were investigated: a group of 30 non-psychiatric healthy controls, 12 participants in an acute state of MDD and 10 participants in remission from MDD (see Table 1 for demographic details).

**Table 1:** **Demographics and clinical variables for groups of healthy (H), depressed (D) and remitted (R) participants**.

	**H (*n* = 30)**	**D (*n* = 12)**	**R (*n* = 10)**	**Statistics**	***P*-value**
Age (mean ± SD)	33.7 ± 11.6	42.0 ± 14.2	37.9 ± 10.1	*F*_(2, 49)_ = 2.1	≥0.1
Gender (% female)	**50**	**50**	**70**	χ^2^_(2)_ = 1.3	≥0.1
IQ^†^	116.6 ± 12.0	109.4 ± 8.5	110.2 ± 10.7	*F*_(2, 44)_ = 2.0	≥0.1
BDl^††^	**n.a.**	28.6 ± 10.3	17.4 ± 4.3	*t*_(8)_ = 2.7	≥0.05^*^
BHS^†††^	4.6 ± 3.9	10.9 ± 4.6	**n.a.**	*t*_(35)_ = 3.7	≥0.001^**^

† Missing values: 1 in the healthy group, 4 in the depressed group.

†† Seven participants in each group.

††† Seven participants in the depressed group.

n.a, not available.

* P < 0.05,

** P < 0.001.

All participants gave their written informed consent before participating in this study. The research project was conducted in accordance with the Declaration of Helsinki and was approved by the local ethics committee. In accordance with ethical guidelines, control participants were financially compensated for their study participation. Healthy study participants were recruited from the Otto-von-Guericke University student body (Magdeburg, Germany) and the local community through newspaper ads and posters displayed throughout the community. General exclusion criteria for the participation in MRI studies involved major medical illnesses, histories of seizures, metallic implants, a history of substance dependence, head trauma with loss of consciousness or pregnancy. Additional exclusion criteria for the control participants included no previous psychiatric history, including no history of affective disorders, as well as no history of neurological or other diseases, as assessed using a custom-made semi-structured clinical questionnaire. Additional exclusion criteria for depressed participants included any psychiatric disorder other than MDD. Depressed participants were taking one or more antidepressants from the following pharmacological classes four participants SSRIs, four NaSSAs and seven participants NARIs/MAOI/others. None of the control subjects were taking any psychotropic medications at the time of the investigation.

Independent groups of depressed and remitted study participants were recruited from either the Department of Psychiatry (University of Magdeburg, Germany) or from the state hospital of Uchtspringe. MDD was diagnosed according to DSM-IV (Diagnostic and Statistical Manual of Mental Disorders, 4th Edition; American Psychiatric Association 1994). Evaluation of acute and remitted stage of MDD was made by the participants' treating psychiatrist. Participants had to be in remitted stage for at least 6 weeks in order to be eligible to undergo an fMRI scan for remitted study participants. In addition, participants were classified as remitted when their Beck Depression Inventory (BDI; Beck et al., 1961) scores improved by at least one level in relation to their score taken during the acute state of depression, e.g., from severe to moderate or from moderate to mild state according to standard BDI cut-off scores. The Hamilton Rating Scale for Depression (HRSD) (Hamilton, 1967) was administered as well.

Depressed affect was further measured in non-psychiatric and depressed individuals using the Beck Hopelessness Scale (BHS; Beck et al., 1974). The BHS is a self-report inventory consisting of 20 true or false statements, which consider the respondents answers for the past week. The questionnaire is considered to mirror more cognitive features of hopelessness like a lack of positive thoughts about the future (“I look forward to the future with hope and enthusiasm.”), a lack of positive expectations (“I don't expect to get what I really want.”) or loss of motivation (“I never get what I want, so it's foolish to want anything.”) (MacLeod et al., 2005; Bjärehed et al., 2010).

Subjective awareness of body perception and interoception was measured using the Body Perception Questionnaire (Porges, 1993). This self-report questionnaire contains four different subscales: the awareness subscale (e.g.: “During most situations I am aware of how hard my heart is beating,” 45 items), stress response (e.g.: “During stressful situations I am aware of breathing more rapidly and shallowly, and having difficulty in catching my breath,” 10 items), autonomic nervous system reactivity (e.g.: “I have difficulty coordinating breathing with talking,” 27 items) and stress style (e.g.: “When I am emotionally stressed because of a specific problem I feel my blood sugar drop,” 12 items). Each item was rated on a five-point Likert scale, ranging from 1 (never) to 5 (always).

### Paradigm

A well-established fMRI paradigm for investigating awareness directed toward intero-/exteroceptive stimuli was implemented here, which has been used previously by our group (Wiebking et al., 2010, 2014a,b). The paradigm consists of three independent awareness conditions (see Figure 4). Each condition was presented 48 times in total in a pseudo-randomized order for 9–13 s each. Participants were instructed to direct their awareness to the external or the internal environment and count corresponding stimuli such as externally applied tones or the own heartbeat. Alternatively, a condition containing no particular task was included, where participants were instructed to disengage and maintain a neutral awareness, i.e., neither directing the awareness to internal nor external stimuli.

In more detail, study participants were made familiar with the fMRI task before the scanning session. Following a standardized protocol, each participant received the same instructions and all had the possibility to practice the paradigm on a computer outside the MRI room. For practice and scanning sessions the software Presentation (Neurobehavioral Systems) was used. The fMRI paradigm utilized dark simple visual stimuli of similar size placed on a light gray background to indicate one of the three condition types. In the scanner, visual stimuli were projected via an LCD projector onto a screen visible through a mirror mounted on the headcoil. During iA conditions, participants were asked to silently count their own heartbeat as long as the task-type indicator–a dark colored heart on a light background–was visible on the screen (jittered between 9 and 13 s). Participants were instructed to focus their awareness on their heartbeat without changing the physical settings (like shifting their position) or any physiological measures (like breathing), which was controlled on-line via the breathing belt of the Siemens Physiological Monitoring Unit (PMU).

During eA, participants were asked to focus on externally applied tones. As long as the task-type indicator–a dark colored musical note on a light background–was visible (jittered between 9 and 13 s) on the screen, study participants counted the number of externally applied tones. Afterwards, the number of counted heartbeats or tones was indicated on a rating scale (4 s). The indicator on the scale was moved by the subject to the labeled position representing the number of beats that they counted. Left and right button presses were used to move the indicator to the left and right side on the scale. This feedback component allowed the monitoring of the subject's attendance to the task.

Auditory stimuli were presented via the scanner loudspeaker. Tones were presented throughout the scanning sessions at an individually adapted volume to match the difficulty of both counting tasks. Thus, study participants had to focus either on internal or external stimuli or to no particular task (see below). In addition, the presentation frequency of the tones was adapted to correspond to each participant's heart-rate. The heart-rate was recorded using the Siemens Physiological Monitoring Unit (PMU) as described previously (Wiebking et al., 2014b). In order to control for habituation effects, the individual onset time of each tone was jittered by 200 ms. Conditions with no particular task (Shulman et al., 2009) were indicated by a dark cross on light background (9 - 13 s). Participants were instructed to disengage, reduce any cognitive work during these periods and maintain an undirected awareness, i.e., focusing neither on internal nor external stimuli. The total experiment consisted of 4 sessions of 9.6 min (1160 volumes in total).

### fMRI data acquisition and analysis

Magnetic resonance imaging was performed on a 3-Tesla MRI system (Siemens Trio, Erlangen, Germany) with a body transmit and eight channel receive head-coil. Functional T2^*^-weighted echo planar images with BOLD contrast were acquired parallel to the AC-PC plane in an odd-even interleaved order. 32 slices per volume were obtained with the following settings: matrix 64 × 64; 32 slices per volume; FoV: 224 × 224 mm^2^; spatial resolution: 3.5 × 3.5 × 4 mm^3^; *T*_*E*_ = 30 ms; *T*_*R*_ = 2000 ms; FA = 80°. T1-weighted anatomical images were also acquired (MPRAGE; 1 × 1 × 2 mm^3^; *T*_*E*_ = 5 ms; *T*_*R*_ = 1650 ms; FA = 7°).

The fMRI images were pre-processed using SPM8 running on MATLAB 7.11 (The Mathworks Inc., Natick, MA, USA). Functional images were slice time corrected with reference to the first acquired slice, corrected for motion artifacts by realignment to the mean functional image, and spatially normalized to the ICBM152 standard space (Ashburner and Friston, 1999). Images were resampled to 2 mm^3^, smoothed with an isotropic 6 mm full-width half-maximum Gaussian kernel and high pass filtered (threshold 128 s).

Since structured noise still remains in the fMRI data after traditional steps of pre-processing, an independent component analysis (ICA) was applied to remove noise and improve the sensitivity and specificity of the results (Beckmann and Smith, 2004). Non-brain voxels were removed using the BET tool in FSL prior to running the ICA (Smith, 2002). Noise and signals of interest were distinguished visually according to a previously described procedure (Wiebking et al., 2014a,b). Participant-level statistical analyses were carried out in SPM8 according to a standard general linear model approach (Friston et al., 1995). All conditions (iA, eA, noTask) were included in the model as separate events, including their feedback phases. The six rigid-body movement parameters calculated per participant during motion correction were included in the model as nuisance variables. Group comparisons were calculated using the FEAT tool in FSL (Smith et al., 2004; Woolrich et al., 2009). In order to control for possible gray or white matter differences as a between-group confounding factor, the individual T1-weighted anatomical images were segmented into gray matter (GM), white matter (WM) and cerebrospinal fluid maps using FAST (Zhang et al., 2001). The resulting partial volume estimates were used to quantify the proportion of GM and WM in each of the regions of interest (left and right insula regions). Individual volume estimates for both GM and WM were then included in the group comparisons as nuisance variables. Group comparisons were masked with the combined regions of interest. An FWE corrected cluster significance level of *P* = 0.05 was used with a Z threshold of 3.1 (Worsley, 2001).

### Definition of insula regions and statistical analysis

**Figure 1:**
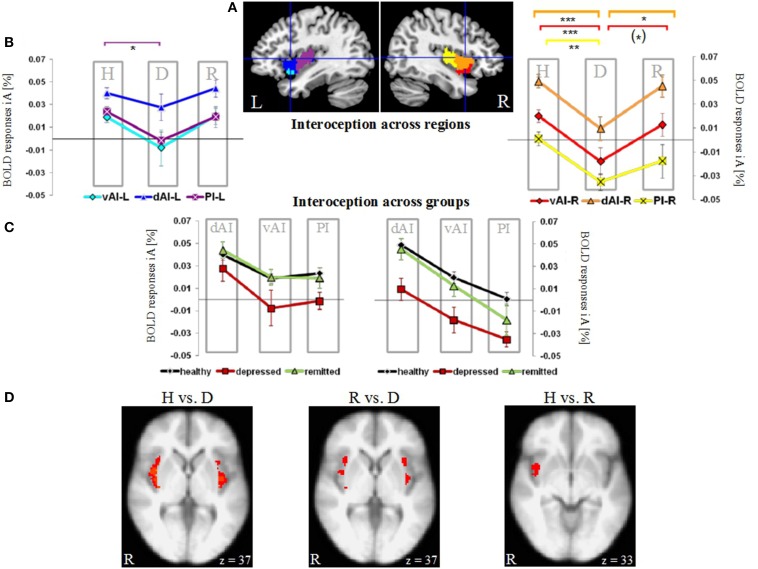
**Neural activity during interoceptive awareness (iA) in the left (L) and right (R) dorsal anterior insula (dAI), ventral anterior insula (vAI) and the posterior insula (PI) in groups of healthy (H), depressed (D) and remitted (R) participants. (A)** Illustration of regions of interest in the left and right insula (L-dAI: blue, L-vAI: cyan, L-PI: purple, R-dAI: orange, R-vAI: red, R-PI: yellow) (provided by Deen et al., 2011). **(B)** Mean iA-related BOLD responses in insula subregions. Data points within columns represent healthy (H), depressed (D) and remitted (R) groups. In the left hemisphere, the PI (purple, square symbols) shows significant differences between healthy and depressed participants. In the right hemisphere, the depressed group differs compared to the healthy group in all regions, whereas differences to the remitted group occur in the dAI (orange, triangular symbols) and vAI (red, diamond symbols). Healthy and remitted groups show no differences. [^***^P < 0.005, ^**^P < 0.01, ^*^P < 0.05, (^*^) *P* < 0.1, *post-hoc* Bonferroni] **(C)** Results shown in **(B)** are re-represented here across groups (healthy: black line, depressed: red line, remitted: green line). Columns represent insula subregions. Healthy and remitted participants show identical neural activation during iA, whilst depressed patients show the lowest activity in each region. Bilateral regions of the dAI show the highest degree of iA-related activity in each study group. In the right hemisphere, this activity linearly decreases with the PI showing the lowest degree of neural activity. **(D)** Voxel-wise results within the bilateral insula comparing the different groups during iA performance (*P* = 0.05 FWE-corrected, Z threshold = 3.1, gray and white matter volumes included as confound). Healthy (left image) and remitted (middle image) participants show increased iA-related BOLD responses compared to depressed participants. Healthy and remitted groups show less extensive differences (right image), with increased BOLD responses in the inferior insula in healthy individuals.

Regions of interest (ROIs) were provided as template images by Ben Deen and colleagues (Deen et al., 2011). They identified three bilateral subregions of the insula based on clustering of functional connectivity patterns: the left (L) and right (R) dorsal anterior to middle insula (dAI), ventral anterior insula (vAI) and the posterior insula (PI) (Figure 1A). This set of insula subdivisions was recently used to further investigate their roles relative to other regions of interest in the brain (Uddin et al., 2013).

**Figure 2:**
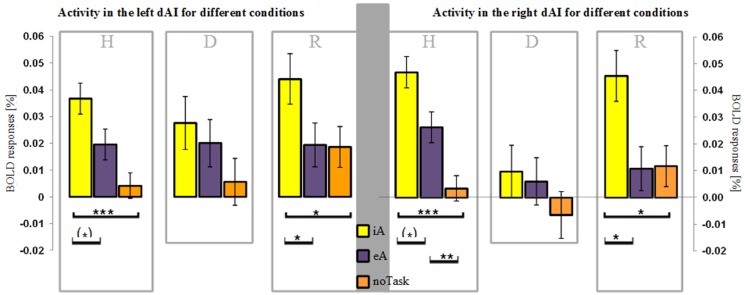
**BOLD responses (mean ± SEM) during different task conditions in healthy (H), depressed (D) and remitted (R) participants in the left and right dorsal anterior insula (dAI).** Interoceptive awareness (iA) is marked in yellow, exteroceptive awareness (eA) is marked in purple and no particular task (noTask) in orange. Healthy and remitted participants show a clear distinction between iA and specifically noTask conditions in both regions. Additionally, iA-related BOLD responses differ to eA-related BOLD responses in both groups and regions, whereas the R-dAI reveals also a differentiation between eA- and noTask-related BOLD responses in the non-psychiatric group. Depressed participants show no differentiation in both regions between any of the three conditions. [^***^P < 0.0005, ^**^P < 0.01, ^*^P < 0.05, (^*^) *P* ≤ 0.1, *post-hoc* Bonferroni].

**Figure 3:**
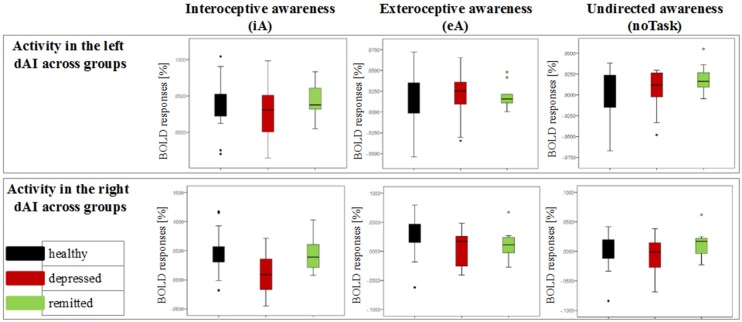
**Detailed overview of the distribution of neural activity in the left and right dorsal anterior insula (dAI) across groups for different conditions (awareness toward internal stimuli on the left side, awareness toward external stimuli in the middle and awareness toward no particular task on the right side).** Black bars indicate the non-psychiatric group, red bars indicate the depressed group and green bars indicate the remitted group.

The MarsBaR (MARSeille Boîte À Région d'Intérêt, http://marsbar.sourceforge.net) toolbox was used to extract task-induced BOLD responses from the voxels within these ROIs. The calculated percent signal change represents an individual value for each participant and each condition within each ROI. Mean percent signal changes (i.e., BOLD responses [%]), which are typically less than 0.1 percent (see MarsBaR documentation), were entered into SPSS 17.0 (SPSS inc., Chicago, IL). Extreme values, which were farther than three interquartile ranges away from the first or third quartile, were defined as outliers and excluded from analysis, which affected single conditions for three healthy participants. To calculate the time course of BOLD responses, MarsBaR's finite impulse response models were used to estimate the response at each time bin. Briefly, the determined values were used to investigate group differences by calculating a multivariate analysis of variance (MANOVA) (Figure 1), to compare BOLD responses of different task conditions (Figure 2), to investigate the distribution of neural activity within subject groups (Figure 3), to calculate time courses of mean BOLD responses across scanning sessions (Figure 5), to perform correlations with subjective hopelessness scores (Figure 6) and to illustrate the time course of BOLD responses (Supplementary Figure 1).

**Figure 4:**
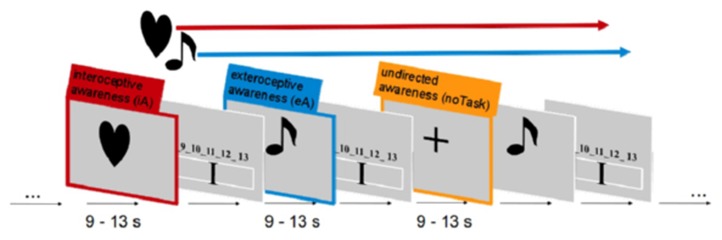
**Visualization of the fMRI paradigm to study interoceptive awareness.** Each condition contained both stimuli types: external tone and internal heartbeat were concurrently ongoing events throughout a scanning session. The different conditions were matched as closely as possible and participants had to direct their awareness either to internal, external or no stimuli.

**Figure 5:**
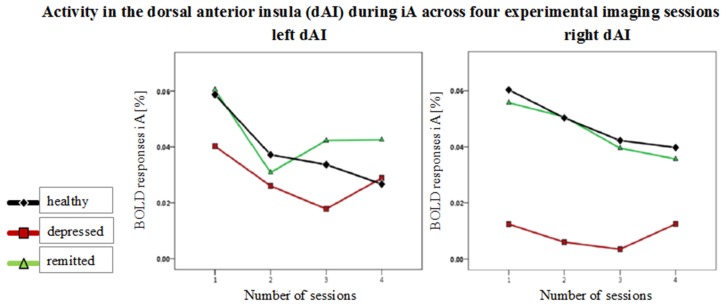
**Mean BOLD responses during interoceptive awareness (iA) across four scanning sessions in the left and right dorsal anterior insula (dAI).** Graphs show responses for healthy (black line, diamond symbols), depressed (red line, squared symbols) and remitted participants (green line, triangular symbols). No group differences occurred over the time course between the four functional scanning sessions.

**Figure 6:**
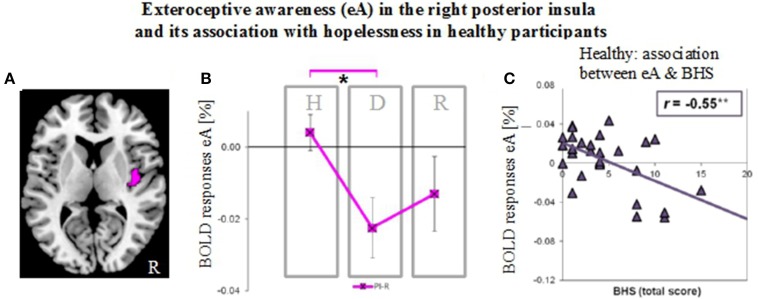
**Neural activity during exteroceptive awareness (eA) in the right posterior insula in groups of healthy (H), depressed (D) and remitted (R) participants and its relation to scores of the Beck Hopelessness Scale (BHS). (A)** Illustration of right posterior insula region of interest (indicated in pink). This region showed significant between-subjects effects during eA as revealed by MANOVA (see Table 2B). **(B)** BOLD responses during eA (mean ± SEM) show differences between healthy and depressed participants in the R-PI (^*^P < 0.05). **(C)** BHS scores correlate negatively (Pearson, two-tailed) with signal changes for eA in healthy participants (^**^P < 0.01).

In more detail, to test neural response differences between the independent study groups, a MANOVA was performed. The three participant groups (*n* = 30 healthy participants, *n* = 12 depressed patients, *n* = 10 participants after remission from MDD) were defined as the between-subjects factor and BOLD responses for each condition (iA, eA, noTask) in each of the six ROIs (dAI, vAI and PI in the left and right hemisphere) were entered as dependent within-subjects variables. Listwise exclusion of three outlier values (see above) led to *n* = 27 healthy participants when calculating the MANOVA. Bonferroni correction was used for *post-hoc* testing in order to reduce type I errors (Figure 1 and Table 2). In order to investigate lateralization effects during iA as indicated by the MANOVA, iA-related BOLD responses in ROIs of each hemisphere were summarized and compared within each group using paired *t*-tests.

**Table 2:**
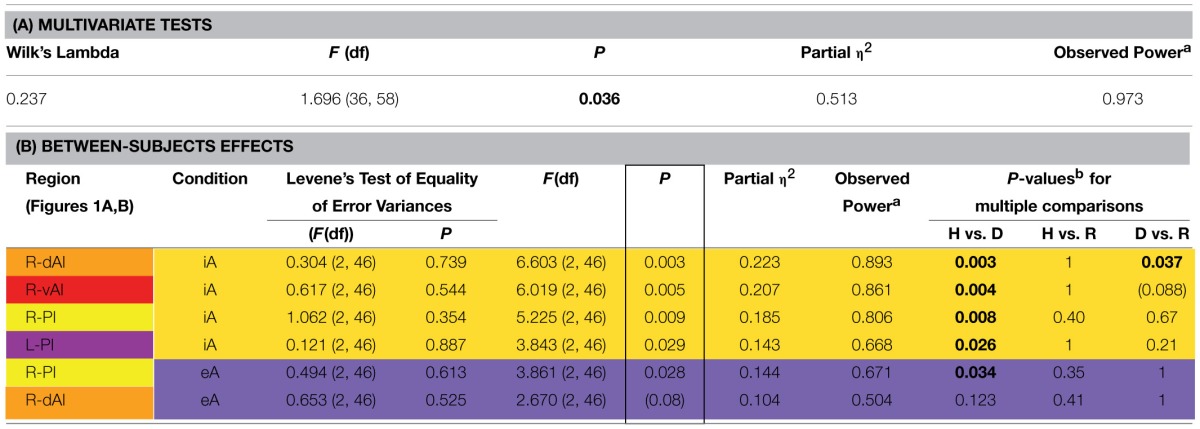
**MANOVA group effects. (A) Results of multivariate tests (including Wilk's Lambda, *F*-value, *P*-value, partial eta squared and observed power based on alpha = 0.05). (B) Results of between-subjects effects (including *F*-value, *P*-value, partial eta squared, observed power based on alpha = 0.05 and *P*-values for multiple comparisons) are sorted according to decreasing *P*-values of significant between-subjects effects (indicated by a box). *P*-values for pair-wise comparisons are based on Bonferroni corrections. The color code for each insula subregion corresponds to the one in Figure 1A. Between-subjects effects regards interoceptive awareness (iA) are highlighted in yellow and between-subjects effects regards exteroceptive awareness (eA) in purple (same color code in Figure 2). Levene's test of equality of variances (at the beginning of B) is not significant, providing assurance that the assumption of homogeneity of variance is not violated**.

^a^*alpha = 0.05*.

^b^P-values are given in bold for significant contrasts (P < 0.05) based on Bonferroni post-hoc tests.

Abbreviations: left (L) and right (R) dorsal anterior insula (dAI), ventral anterior insula (vAl), posterior insula (PI), healthy (H) participants, depressed (D) patients, remitted (R) patients. Note that single values are excluded listwise when calculating MANOVA, resulting in n = 27 healthy participants.

As the dAI showed the most significant group effect and the highest degree of iA-related BOLD responses, the relationship between different task conditions (iA, eA, noTask) in each group (healthy, depressed and remitted participants) was investigated. Differences within the R-dAI and L-dAI were identified using the three task conditions as between-subjects factor and the BOLD responses of each group as within-subjects variables. *Post-hoc* tests were Bonferroni corrected. In a final step, BOLD responses in regions showing significant between-subjects effects (as assessed using MANOVA) were correlated (Pearson, two-tailed) with scores of the Beck Hopelessness Scale (BHS; Beck et al., 1974).

## Results

### Participants

An analysis of variance (ANOVA) showed no differences between the three study groups for age, gender or intelligence (Table 1). BDI scores differed significantly (*P* ≤ 0.05) between independent groups of depressed and remitted participants, but were not collected for healthy due to expected low statistical spread within this group. Mean scores of the HRSD for eight depressed participants were 17.38 (± 8.03). BHS scores were not available for remitted participants and differed significantly (*P* ≤ 0.001) between non-psychiatric and depressed participants.

### Results across insula regions (Figure 1, Table 2)

The MANOVA across groups, regions and conditions revealed a significant effect for group [*F*_(hypothesis_ *df*: 36, error *df*: 58) = 1.696, *P* = 0.036; Wilk's Lambda = 0.237; partial η^2^ = 0.513; observed power = 0.973 at alpha 0.05; please see Table 2A]. As detailed in Table 2B, significant between-subjects effects occurred mainly during iA conditions in the R-dAI [*F*_(2, 46)_ = 6.603; *P* = 0.003; partial η^2^ = 0.223; observed power = 0.893], L-PI [*F*_(2, 46)_ = 3.843; *P* = 0.029; partial η^2^ = 0.143; observed power = 0.668], R-PI [*F*_(2, 46)_ = 5.225; *P* = 0.009; partial η^2^ = 0.185; observed power = 0.806] and R-vAI [*F*_(2, 46)_ = 6.019; *P* = 0.005; partial η^2^ = 0.207; observed power = 0.861]. EA conditions, on the other hand, showed a single significant main group difference in the R-PI [*F*_(2, 46)_ = 3.861; *P* = 0.028; partial η^2^ = 0.144; observed power = 0.671] and a marginal effect in the R-dAI [*F*_(2, 46)_ = 2.670; *P* = 0.08; partial η^2^ = 0.104; observed power = 0.504]. No group effects occurred during noTask conditions. Levene's test of equality of error variances (Table 2B) showed no significant results (ranging from *P* = 0.354–0.887), indicating similar error variances in each of the three subject groups.

Figure 1A illustrates the regions of interest provided by Deen et al. (Deen et al., 2011). The color code of each region is used to visualize the results of the Bonferroni *post-hoc* tests in Figure 1B. The results of the Bonferroni *post-hoc* tests, used to determine which groups differ from each other in which region, are detailed in Table 2B and illustrated in Figure 1B (interoception across regions). Neural activity during iA in the R-dAI of depressed individuals (mean ± SD: 0.01% ± 0.034, *n* = 12) was significantly lower compared to both healthy (0.049% ± 0.032, *n* = 27, *P* = 0.003) and remitted participants (0.045% ± 0.030,*n* = 10, *P* = 0.037) (orange line, Figure 1B). There were no statistically significant differences between healthy and remitted participants (*P* = 1, see also Table 2B).

A similar pattern of neural responses during performance of the iA task occurred in the R-vAI (red line, Figure 1B): BOLD responses during iA in depressed patients (−0.018% ± 0.040) were significantly reduced compared to those in healthy (0.020% ± 0.028, *P* = 0.004) and marginally reduced compared to remitted participants (0.013% ± 0.030, *P* = 0.088). Values between healthy and remitted participants did not differ (*P* = 1, see also Table 3 detailing mean BOLD responses in insula subregions showing significant between-subjects effects as revealed by MANOVA).

**Table 3:**
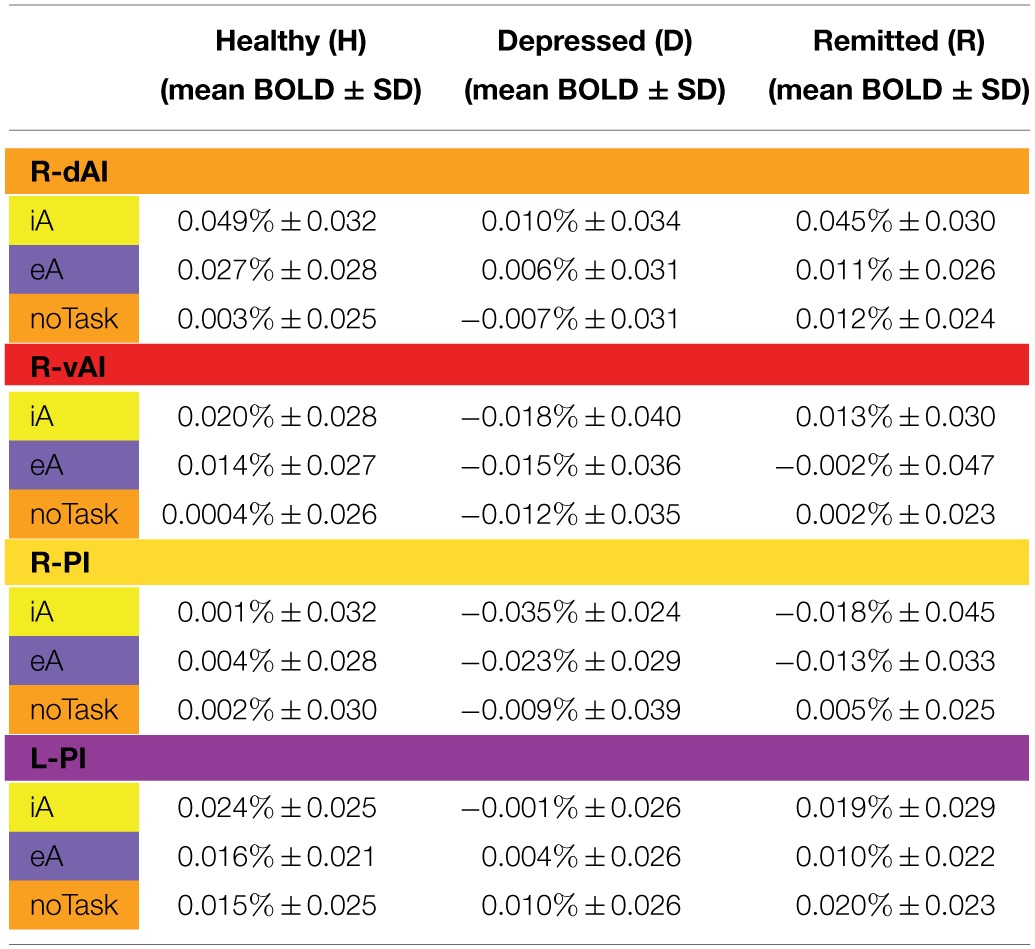
**BOLD responses (mean % ± SD) for healthy (*n* = 27), depressed (*n* = 12) and remitted participants (*n* = 10) in regions showing significant between-subjects effects during iA or eA as revealed by MANOVA (Table 2B)**.

iA: interoceptive awareness; R-dAl: right dorsal anterior insula; eA: exteroceptive awareness; R-vAl: right ventral anterior insula; noTask: undirected awareness; R-Pl/L-Pl: right/ left posterior insula.

The color code for each insula subregion corresponds to the one introduced in Figure 1A. The color code for each condition (iA, eA, noTask) corresponds to the one introduced in Figure 2.

In addition, depressed showed significant differences compared to healthy participants in the bilateral PI during iA (left hemisphere, purple: *P* = 0.026; right hemisphere, yellow: *P* = 0.008). Depressed participants showed lower BOLD responses in the PI (L: −0.001% ± 0.026; R: −0.035% ± 0.024) compared to healthy (L: 0.024% ± 0.025; R: 0.001% ± 0.032). No significant differences existed between remitted (L: 0.019% ± 0.029; R: −0.018% ± 0.045) and healthy (L: *P* = 1; R: *P* = 0.395), nor between remitted and depressed patients in the PI (L: *P* = 0.214; R: *P* = 0.665). The purple line in Figure 1B illustrates the results for the L-PI; the yellow line illustrates results for the R-PI. The two remaining regions on the left side, the dAI (blue line, Figure 1B) and the vAI (cyan line, Figure 1B), showed no significant group differences.

In summary, predominantly iA-related BOLD responses in insula subregions of the right hemisphere showed significant group differences when comparing depressed participants to both remitted as well as healthy (Table 2B). This lateralization effect was confirmed by comparing summarized mean BOLD responses during iA in the left vs. right hemisphere (independent of insula subregion) using a paired *t*-test: neural activity during iA differed between left and right ROIs in depressed patients [*t*_(70)_ = 2.151, *P* = 0.035], with significantly lower activity on the right side (R: −0.015% ± 0.04, L: 0.006% ± 0.04). No differences were seen in healthy [*t*_(163)_ = 0.652, *P* = 0.516] or remitted participants [*t*_(49)_ = 1.526, *P* = 0.134].

By illustrating BOLD responses during iA across different subject groups (Figure 1C) rather than across regions (as done in Figure 1B), it was further underlined that healthy (black line, Figure 1C) and remitted participants (green line) showed identical neural activation levels during iA within the different insula regions. This holds true for the left as well as for the right hemisphere. Moreover, the visualization demonstrated that the degree of neural iA-related activity in the right insula continuously decreased from the dAI to the PI in each study group. Referring to this pattern of decreasing iA activity, the neural activity during iA in all three independent subject groups in each hemisphere displayed an identical degree of reduction from the dAI to the PI. Other results, which can be inferred from Figure 1B as well, relate to the depressed study group (red line), which showed the lowest levels of activity in each insula subregion. The dAI showed the highest BOLD responses during iA in each study group.

Having shown group differences mainly during iA-related neural activity in *a priori* defined regions of interest, voxel-wise comparisons within the bilateral insula as a whole were used to further illustrate the anatomical localization of group differences (Figure 1D). Healthy participants showed increased neural activity during iA when compared to depressed patients in the majority of the bilateral insula (Figure 1D, brain image on left side). Remitted participants showed higher neural activity during iA when compared to depressed patients in the bilateral insula, but to a lesser spatial extent (middle image of Figure 1D). Comparisons between non-psychiatric and remitted participants were less evident and revealed increased neural activity in healthy in more inferior regions of the insula (Figure 1D, right side).

### Results in the dAI (Figure 2)

Following our hypothesis of an impaired response differentiation between stimuli types in depressed participants in regions sensitive to iA, we investigated the BOLD responses during iA, eA and noTask in the bilateral dAI. This region was chosen for two reasons: firstly, the dAI showed the highest BOLD responses during iA in all subject groups (Figure 1C), indicating a high sensitivity to iA. Secondly, depressed patients showed the greatest differences compared to healthy and remitted participants in the R-dAI (Figure 1B, orange line).

Depressed patients showed no differentiation between different stimuli types in the right or left dAI [L-dAI: *F*_(2, 33)_ = 1.459, *P* = 0.247, R-dAI: *F*_(2, 33)_ = 0.853, *P* = 0.435] (Figure 2). In contrast, differentiations between different stimuli types within the dAI occurred in healthy [L-dAI: *F*_(2, 86)_ = 8.765, *P* < 0.0005, R-dAI: *F*_(2, 86)_ = 14.166, *P* < 0.00001] and remitted participants [L-dAI: *F*_(2, 27)_ = 5.777, *P* = 0.008, R-dAI: *F*_(2, 27)_ = 5.408, *P* = 0.011].

Bonferroni *post-hoc* tests revealed the same pattern of differentiation between different stimuli types in the L-dAI for healthy and remitted participants. As such, iA differed to eA (healthy: *P* = 0.088, remitted: *P* = 0.022) as well as to noTask (healthy: *P* = 0.0005, remitted: *P* = 0.018), whilst eA and noTask showed no differences (healthy: *P* = 0.15, remitted: *P* = 1). Descriptive statistics showed the most positive values for iA-related BOLD responses (healthy: 0.037% ± 0.029; remitted: 0.044% ± 0.023) and the least for noTask (healthy: 0.004% ± 0.026; remitted: 0.019% ± 0.018) (left side of Figures 2, 3)^1^. The same differentiation of iA compared to eA and noTask occurred in both healthy and remitted participants in the R-dAI. In detail, iA-related BOLD responses differed to eA (healthy: *P* = 0.094, remitted: *P* = 0.023) as well as to BOLD responses for the noTask condition (healthy: *P* = 0.0005, remitted: *P* = 0.028). Healthy showed an additional difference between eA and noTask (*P* = 0.009). Again, iA-related BOLD responses showed the most positive values (healthy: 0.045% ± 0.033; remitted: 0.045% ± 0.030) and the least estimates for noTask (healthy: 0.003% ± 0.035; remitted: 0.012% ± 0.024) (right side of Figures 2, 3).

No group differences were observed over the time course of the four functional sessions in the different insula regions [*F*_(hypothesis *df*: 6, error *df*: 88)_ = 0.39, *P* = 0.88; Wilk's Lambda = 0.95, partial η^2^ = 0.026]. Figure 5 shows mean BOLD responses during iA across the four scanning sessions in the left and right dAI. BOLD time courses (0 - 10 s) for each group in regions showing between-subjects effects are shown in Supplementary Figure 1.

### Results in the PI (Figure 6) and associations with hopelessness

As established by MANOVA results, only one significant between-subjects effects occurred during eA in the right PI (Table 2B, region illustrated in Figure 6A). Bonferroni *post-hoc* tests revealed lower BOLD responses in the depressed group (−0.023% ± 0.029) when compared to the non-psychiatric study group (0.004% ± 0.028, *P* = 0.034) (Figure 6B and Table 3). No significant differences were seen between remitted (−0.013% ± 0.033) and healthy (*P* = 0.352) or depressed participants (*P* = 1).

Finally, BOLD responses during iA and eA in regions showing significant between-subjects effects (Table 2B) were correlated with subjective measures of hopelessness (BHS) (Table 4). Although separate groups of healthy and depressed participants showed no association between iA-related BOLD responses and BHS in the respective regions (R-dAI, R-vAI, right/left PI), a combined group of healthy and depressed participants revealed additional associations between iA and BHS in each of those regions. Neural activity regards the above mentioned between-subjects effect for eA in the right PI, on the other side, was negatively correlated with BHS scores in the healthy group (see also Figure 6C).

**Table 4:**
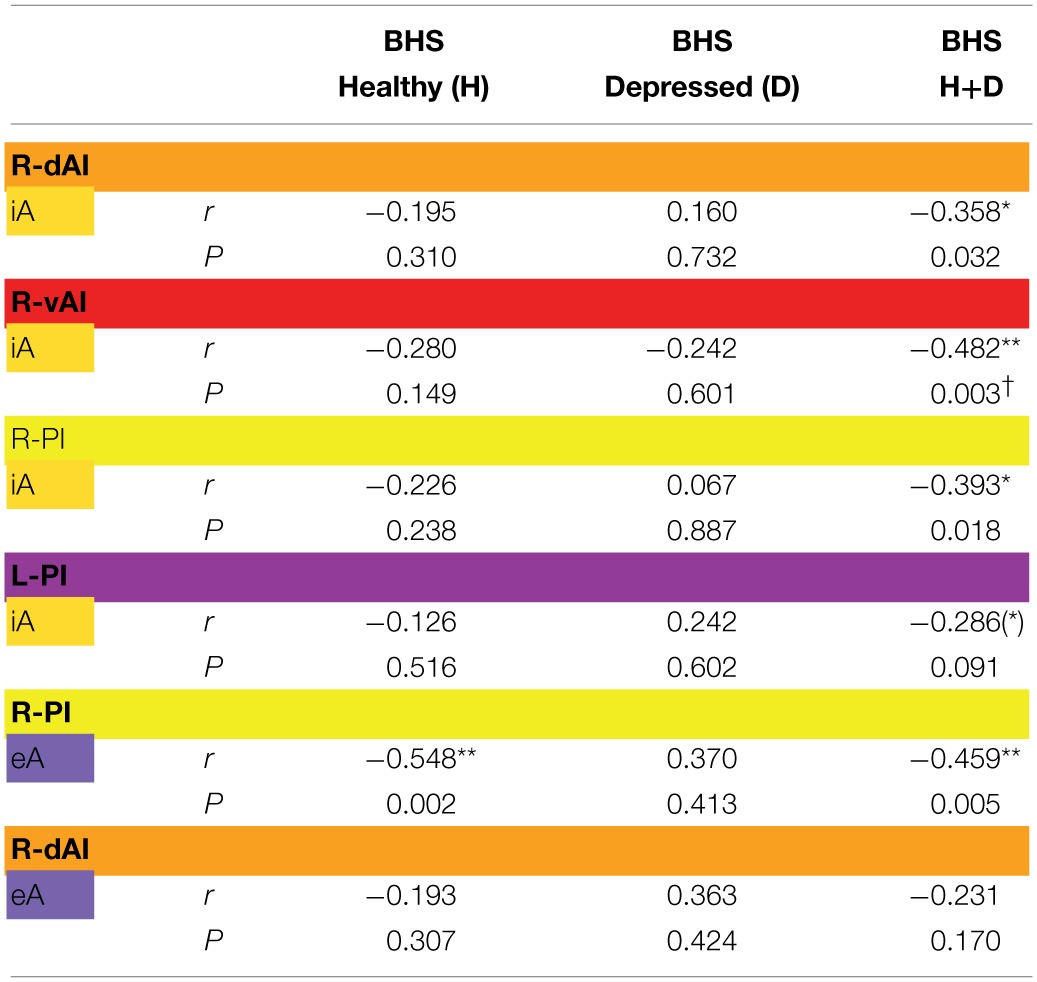
**Correlations (Pearson *r*-values, two-tailed) between signal changes in regions showing significant between-subjects as revealed by MANOVA (Table 1) and scores of the Beck Hopelessness Scale (BHS)**.

^**^P ≤ 0.005, ^*^P < 0.05, (^*^)P < 0.1.

†please see also Supplementary Figure 2.

Columns show results for n = 30 healthy participants (H), n = 7 depressed patients (D) and a combined group of healthy and depressed participants (H + D, n = 37). BHS scores for the remitted patient group were not collected.

The color code for each insula subregion corresponds to the one introduced in Figure 1A. According to the color code in Figure 2, results referring to interoceptive awareness (iA) are highlighted in yellow and results referring to exteroceptive awareness (eA) in purple.

## Discussion

Using *a priori* subregions of the insula (Deen et al., 2011), neural activity during iA, eA and noTask periods was investigated in independent study groups consisting of non-psychiatric controls, depressed and remitted participants. Whilst BOLD activity in response to IA or other conditions did not differ between healthy and remitted participants, reduced iA activity in MDD patients was the main factor differentiating them from healthy and remitted participants. This finding is in concordance with our hypothesis of a neural deficit in response to iA in MDD. Moreover, MDD patients show in contrast to healthy and remitted participants reduced differentiations between neural signals in iA sensitive regions. These preliminary results show for the first time deficits in neural processing of iA in anterior insula regions in MDD and a regeneration of neural activity in remission from depression in an independent participant group. Our findings have potential novel implications for both diagnosis and therapy of MDD.

Prior results in non-psychiatric individuals support the idea of the insula acting as a bridging region between internal and external awareness (Farb et al., 2013; Simmons et al., 2013; Paul et al., 2013). Nevertheless, anterior regions particularly in the right hemisphere are often reported to be more engaged in heartbeat monitoring (i.e., iA) tasks, showing higher BOLD responses (Critchley et al., 2004; Zaki et al., 2012). This finding can be confirmed and expanded upon in this study. Here, the dAI shows the highest level of activity during iA across regions and study groups. Activity levels particularly in the right dAI then decrease linearly toward the more posterior regions of the insula. This finding is well in accordance with a recent study using the same set of insula subregions and describing the “nuanced functional profiles of insular subregions, [whilst specifically the] dorsal anterior insula can be considered a critical functional hub in the human brain” (Uddin et al., 2013). In the depressed group, iA activity in the right insula is particularly reduced, showing a significantly lower BOLD response amplitude compared to left insula regions. The shape of the mean BOLD responses across scanning sessions does not differ between groups (Figure 5), meaning that a confounding effect of response shape (rather than amplitude) can be excluded. The particular effect difference in the right insula is consistent with previous lateralization findings (Kotani et al., 2009), with hypotheses that posit a major role for the right insula in iA (Craig, 2002, 2003, 2004), and with findings of distinct network connectivity in the right dAI and vAI (Touroutoglou et al., 2012).

Interestingly, the general pattern of insula activation across the different subregions is similar in all three study groups (Figure 1C). This suggests that the iA-related processing deficit seen in MDD is not restricted to a particular portion of the insula but is instead a general hypo-response across the different anatomical and functional subregions of the insula. This is in accordance with suggested abnormalities in amino acid neurotransmitter function and impaired energy metabolism in MDD (Abdallah et al., 2014), which might argue for a global brain mechanism underlying the observed neural hypo-responses, as distinguished from neural responses to specific content. Most importantly, the neural hypo-response during iA performance that is seen in patients suffering from depression is not observable in the other two independent participant groups. The neural activity during iA in the insula seen in patients recovered from major depression is very similar to the neural activity during iA seen in non-psychiatric participants, indicating that the neural insula responses during iA might serve as a state marker of MDD. However, deficits in energy metabolism in major depression and its potential normalization after remission need to be included in future studies as an additional variable, e.g., by using ^13^C MRS (magnetic resonance spectroscopy) to provide additional information about glucose dynamics. By this, it will be possible to further unravel general from specific deficits underlying the pathophysiology of MDD.

As well as a general insula hypo-response, there is a lack of stimulus differentiation in anterior insula regions in MDD. In contrast, healthy and remitted participants show a clear differentiation between the different stimulus types (iA, eA and noTask) in these regions. This differentiation was made even though both the heartbeat and the tone stimuli were continuously present during all trials. In depressed participants the different stimuli produce comparable neural responses; if a role of the insula is to integrate intero-/exteroceptive stimuli for homeostatic purposes and in doing so produce a sense of material self (Craig, 2002), then this lack of differentiation may lead to an altered self-awareness in depression. Such an effect would be consistent with the observed abnormal bodily awareness in heautoscopy that has been linked to iA processing in the insula (Heydrich and Blanke, 2013). Finally, such a dis-integration of intero-/exteroceptive stimuli in MDD can be related to the interoceptive predictive coding model proposed by Paulus and Stein (Paulus and Stein, 2010), according to which noisy afferent input in the insula in depression leads to an interoceptive prediction error. Both findings–non-differential responses to different stimulus types and a general insula hypo-metabolism–may provide evidence for such a coding error where insula stimulus responses are too unspecific during acute MDD.

Regards eA-related BOLD responses, only one significant between-subjects effects occurred in the right PI. Depressed participants showed decreased neural activity as compared to non-psychiatric controls, which may reflect altered awareness of the environment that is reported in MDD (Grimm et al., 2009; Wiebking et al., 2010; Northoff et al., 2011). A strong relationship between eA and BHS scores in healthy individuals supports this suggestion (increased hopelessness is associated with reduced eA activity). Decreased activity in the PI–a region that has anatomical connections to the auditory cortex (Cloutman et al., 2012) and is involved in basic interoceptive (Craig, 2002; Deen et al., 2011) and auditory processes (Bamiou et al., 2003; Cloutman et al., 2012)–might be an indicator that environmental stimuli cannot be integrated with interoceptive states in MDD (Critchley, 2009; Sliz and Hayley, 2012). In regions showing significant between-subjects effects of iA-related BOLD responses there was no correlation with BHS scores in the depressed or healthy group alone, but a combined group of depressed and healthy (mirroring a group with higher variances) showed significant negative correlations in each region that was identified by means of MANOVA (Table 4). In conjunction with the insula hypo-responsiveness in MDD, this suggests that depressed individuals can be considered to be at the lower extreme end of a continuous relationship between interoception, depression and hopelessness.

The insula, along with the dorsal anterior cingulate cortex (dACC) and superior temporal lobe, forms part of the so-called salience network (Seeley et al., 2007). This network is thought to be involved in the coordination of behavioral responses (Medford and Critchley, 2010; Menon and Uddin, 2010) through the detection of and orientation to subjectively relevant internal or external stimuli (Seeley et al., 2007). In MDD, intrinsic functional connectivity between the network's constituent regions has been reported to be reduced (Hamilton et al., 2012; Sliz and Hayley, 2012; Manoliu et al., 2013; Belleau et al., 2014; Yuen et al., 2014). The right anterior insula in particular appears to display altered connectivity in MDD (Manoliu et al., 2013), which corresponds with the altered responses in that region reported here. As well as connectivity within the salience network, connectivity between it and other brain networks—specifically the default mode network (DMN) and the executive control network—is also altered in MDD (Manoliu et al., 2013; Belleau et al., 2014). This in turn has been linked to a pathological preponderance of DMN activity over that in the salience and executive control networks (Hamilton et al., 2012; Manoliu et al., 2013). The overall blunting of task responses in MDD seen here, along with the lack of differentiation between the stimulus types, is thus suggestive of a network interaction dysfunction influencing the task-specific responses. This possibility remains to be investigated in the context of iA, however, as does the directionality of any interactions (i.e., salience network to DMN or vice versa; although it may be noted that initial evidence suggests that the former may be the case (Ham et al., 2013).

Several limitations of the study may be considered. Although the sample sizes exceed similar studies investigating pre- and post-depression in fMRI (Schaefer et al., 2006), the observed power of the MANOVA indicates robustness and the statistical power of the fMRI paradigm is excellent (48 repetitions per condition), the current results must be treated as preliminary findings. Confirmation through a larger study containing equal sample sizes would therefore be worthwhile. A second potential limitation is that depressed patients were medicated. That this factor could be driving the results observed is unlikely, however, given that remitted participants were also medicated. Despite this, future studies may wish to include an unmedicated depressed group to confirm the effects seen here, given that a medication effect in comparison to non-psychiatric controls cannot be excluded. Finally, inclusion of additional behavioral measurements will help increase the explanatory power of future fMRI studies investigating iA. These may contain longer heartbeat counting phases during fMRI and ECG (electrocardiogram) measurements during fMRI as well as outside the scanner in order to obtain robust measurements of task accuracy. The interpretation of interoceptive accuracy is difficult, however, as previous studies pointed out unreliable results in non-psychiatric controls (Willem Van der Does et al., 2000), inconsistent findings in depression (Dunn et al., 2007; Pollatos et al., 2009; Furman et al., 2013) and even opposite effects in anxiety patients (Willem Van der Does et al., 2000; Domschke et al., 2010). Given the high prevalence of depressive/anxiety comorbidity and the influence of anxiety traits on insula activity (Terasawa et al., 2013), this needs to be further disentangled in future studies and may be seen as limitation factor of the current study as well, as no clinical or psychometric measures for anxiety were applied. It needs to be pointed out that the current study did not aim to investigate heartbeat accuracy (see Garfinkel et al., 2014 for conceptualization), but rather the neural activity changes due to focal awareness shift directed toward intero-/exteroceptive stimuli, respectively (see also Avery et al., 2014).

In conclusion, we show an insula hypo-response in MDD, particularly during iA conditions in comparison to remitted and non-psychiatric participants. This effect is no longer present after remission from depression, implying a flexible mechanism. Since all participant groups showed a comparable activity pattern across the insula subregions, aberrant IA-related BOLD responses in depressed patients can be traced back to an overall attenuation of activity. Supporting a hypothesis of altered processing of interoceptive afferents in depression (Paulus and Stein, 2010), the MDD group showed no differentiation between different stimulus types in iA-sensitive regions. Given this, our results have potential implications for the feasibility of using interoception-related neurofeedback to help speed recovery in MDD by normalizing insula activity (Wiebking and Northoff, 2014). These therapies may also serve as preventative strategies for individuals in remitted states following depression.

### Conflict of interest statement

The authors declare that the research was conducted in the absence of any commercial or financial relationships that could be construed as a potential conflict of interest.

## Footnotes

^1^Please note that listwise exclusion of three outliers lead to n = 27 healthy participants in case of the MANOVA (Figure 1, Table 3), which is not the case when calculating an ANOVA. Hence, mean values for healthy participants may vary slightly depending on the underlying statistical test.

## Supplementary material

Supplementary Figure 1 **BOLD responses (% mean ± SEM) per time bin (TR = 2000 ms) in each of the six regions of interest (please refer also to Figure 1A) for each condition (iA, eA, noTask) in each subject group (healthy, depressed and remitted participants)**. The border of each chart serves to identify the insula region (cyan: left ventral anterior insula, blue: left dorsal anterior insula, purple: left posterior insula, red: right ventral anterior insula, orange: right dorsal anterior insula, yellow: right posterior insula). Corresponding to the color code introduced in Figure 2, interoceptive awareness is marked in yellow, exteroceptive awareness is marked in purple and no particular task in orange. Group-specific charts are arranged vertically. Please note different intervals of *y*-axis.

Supplementary Figure 2 **(A)** Mean total scores of the Beck Hopelessness Scale (BHS), Body Perception Questionnaire (BPQ) subscale awareness of autonomic nervous system reactivity (ANSR) and stress style (BPQ-stressStyle). Depressed patients score significantly higher on each questionnaire (see also Supplementary Table 1). **(B,C)** Correlation (Pearson, two-tailed) between awareness of autonomic nervous system reactivity (ANSR) and BHS **(B)** and BOLD responses during iA in the R-vAI **(C)** for a combined group of healthy and depressed participants (see also Supplementary Table 1, *n* = 35). **(D)** Correlation (Pearson, two-tailed) between BOLD responses during iA in the R-vAI and BHS for a combined group of healthy and depressed participants (see also Table 4). **(E,F)** Correlation (Pearson, two-tailed) between BPQ's subscale for stress style and BHS **(E)** and BOLD responses during iA in the R-vAI **(F)** for a combined group of healthy and depressed participants (see also Supplementary Table 1).

Supplementary Table 1 **Significantly different BPQ (Body Perception Questionnaire) scores between healthy (*n* = 30) and depressed participants (*n* = 6) (upper part of table)**. Correlation (Pearson, two-tailed) between BPQ and scores of the Beck Hopelessness Scale (BHS) for healthy, depressed and a combined group (middle part of table). Correlation (Pearson, two-tailed) between BPQ and BOLD responses in regions showing significant group differences according to MANOVA (Table 1).

## Abbreviations

BHS: Beck Hopelessness Scale
BOLD: blood oxygen level dependent
D: depressed participants
(L/R)-dAI: left/right dorsal anterior insula
eA: exteroceptive awareness
fMRI: functional magnetic resonance imaging
H: healthy participants
iA: interoceptive awareness
MANOVA: multivariate analysis of variance
MDD: major depressive disorder
(L/R)-PI: posterior insula
R: remitted participants
ROI: region of interest
(L/R)-vAI: ventral anterior insula.
