# Supplementary figures and images for: Interoception in insula subregions as a possible state marker for depression—an exploratory fMRI study investigating healthy, depressed and remitted participants
Source: Front Behav Neurosci. 2015 Apr 10;9:82. doi: 10.3389/fnbeh.2015.00082 (PMC4392695; doi:10.3389/fnbeh.2015.00082)

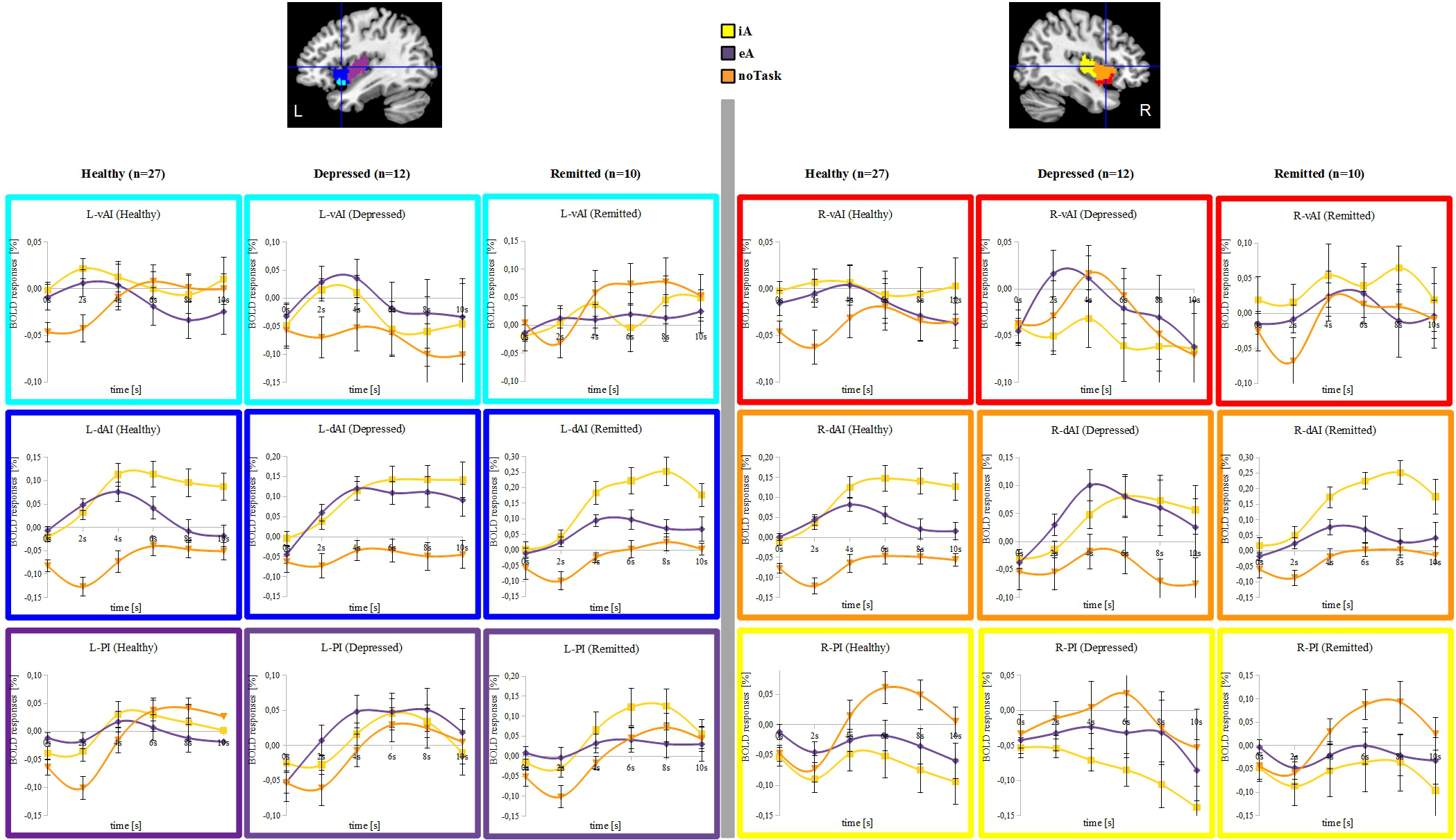

Supplement: Supplementary Figure 1 — BOLD responses (% mean ± SEM) per time bin (TR = 2000 ms) in each of the six regions of interest (please refer also to Figure 1A) for each condition (iA, eA, noTask) in each subject group (healthy, depressed and remitted participants). The border of each chart serves to identify the insula region (cyan: left ventral anterior insula, blue: left dorsal anterior insula, purple: left posterior insula, red: right ventral anterior insula, orange: right dorsal anterior insula, yellow: right posterior insula). Corresponding to the color code introduced in Figure 2, interoceptive awareness is marked in yellow, exteroceptive awareness is marked in purple and no particular task in orange. Group-specific charts are arranged vertically. Please note different intervals of y-axis. [file Image1.TIF]

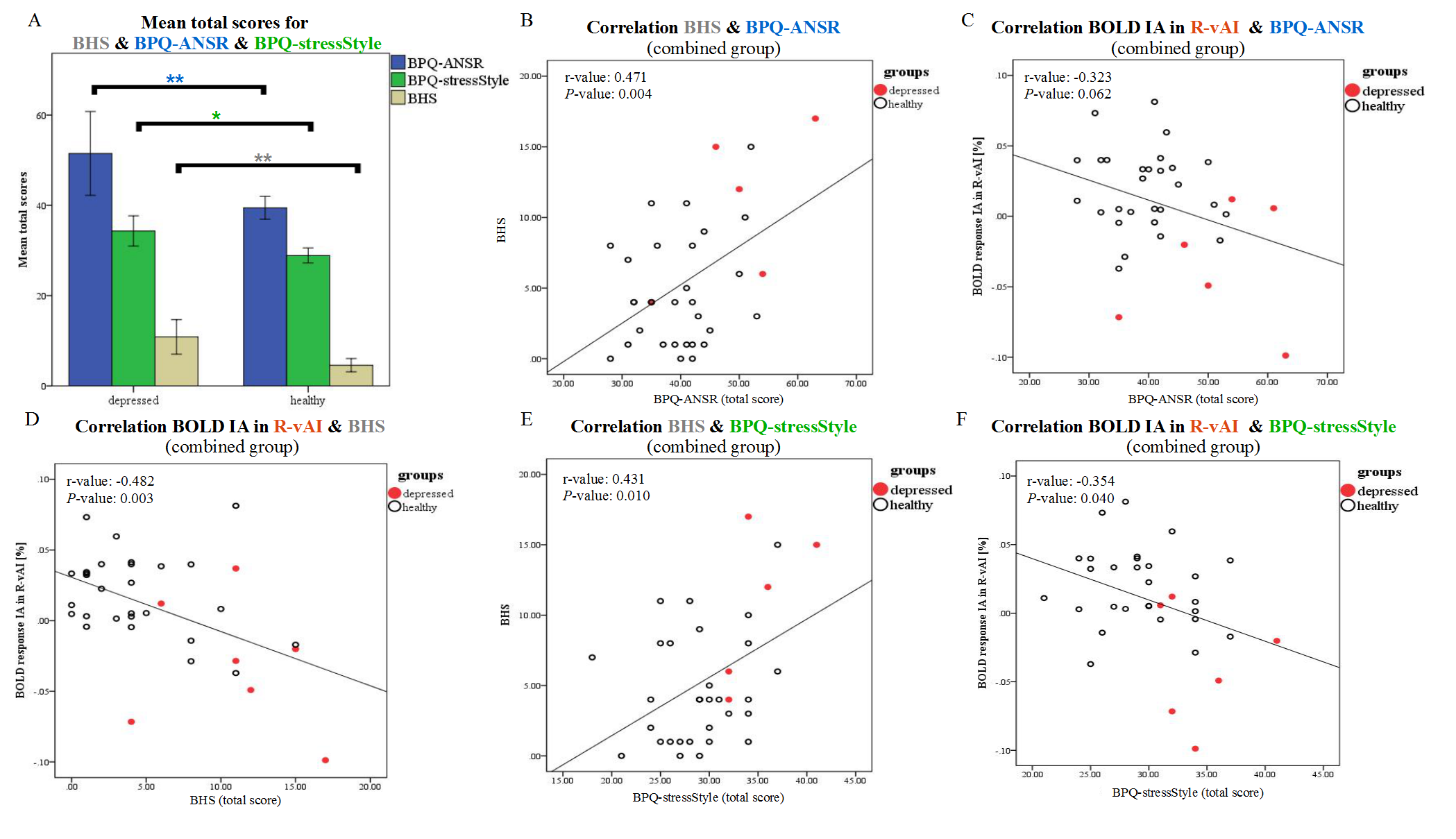

Supplement: Supplementary Figure 2 — (A) Mean total scores of the Beck Hopelessness Scale (BHS), Body Perception Questionnaire (BPQ) subscale awareness of autonomic nervous system reactivity (ANSR) and stress style (BPQ-stressStyle). Depressed patients score significantly higher on each questionnaire (see also Supplementary Table 1). (B,C) Correlation (Pearson, two-tailed) between awareness of autonomic nervous system reactivity (ANSR) and BHS (B) and BOLD responses during iA in the R-vAI (C) for a combined group of healthy and depressed participants (see also Supplementary Table 1, n = 35). (D) Correlation (Pearson, two-tailed) between BOLD responses during iA in the R-vAI and BHS for a combined group of healthy and depressed participants (see also Table 4). (E,F) Correlation (Pearson, two-tailed) between BPQ's subscale for stress style and BHS (E) and BOLD responses during iA in the R-vAI (F) for a combined group of healthy and depressed participants (see also Supplementary Table 1). [file Image2.TIF]
