# Supplementary material for: Interoception in insula subregions as a possible state marker for depression—an exploratory fMRI study investigating healthy, depressed and remitted participants
Source: Front Behav Neurosci. 2015 Apr 10;9:82. doi: 10.3389/fnbeh.2015.00082 (PMC4392695; doi:10.3389/fnbeh.2015.00082)
Supplement: Supplementary Table 1 — Significantly different BPQ (Body Perception Questionnaire) scores between healthy (n = 30) and depressed participants (n = 6) (upper part of table). Correlation (Pearson, two-tailed) between BPQ and scores of the Beck Hopelessness Scale (BHS) for healthy, depressed and a combined group (middle part of table). Correlation (Pearson, two-tailed) between BPQ and BOLD responses in regions showing significant group differences according to MANOVA (Table 1). [file Table1.PDF]

**T-test BPQ**		**BPQ-total**	**BPQ-stressResp**	**BPQ-ANSR**	**BPQ-stressStyle**
		**t(df); *P*-value**	**t(df); *P*-value**	**t(df); *P*-value**	**t(df); *P*-value**
**Healthy**	**(mean ± SD)**	199.033 ± 50.243	2.287 ± 7.114	1.462 ± 0.252	2.408 ± 0.373
**Depressed**	**(mean ± SD)**	249.500 ± 65.705	3.033 ± 1.031	1.907 ± 0.383	2.861 ± 0.310
**Statistics**		*t*(34) = -2.137; **0.040**	*t*(34) = -2.178; **0.036**	*t*(34) = -3.625; **0.001**[†]	*t*(34) = -2.779; **0.009**[†]

**Correlation BPQ & BHS**		**BPQ-total**	**BPQ-stressResp**	**BPQ-ANSR**	**BPQ-stressStyle**
		**(r-value, *P*-value)**	**(r-value, *P*-value)**	**(r-value, *P*-value)**	**(r-value, *P*-value)**
**BHS**	**healthy (H)**	0.046, 0.810	0.120, 0.529	0.238, 0.205	0.222, 0.238
**BHS**	**depressed (D)**	0.234, 0.694	0.499, 0.392	0.631, 0.253	0.653, 0.232
**BHS**	**H+D**	0.237, 0.170	0.338, **0.047**	0.471, **0.004**[†]	0.431, **0.010**[†]

**Correlation BPQ & BOLD**			**BPQ-total**	**BPQ-stressResp**	**BPQ-ANSR**	**BPQ-stressStyle**
			**(r-value, *P*-value)**	**(r-value, *P*-value)**	**(r-value, *P*-value)**	**(r-value, *P*-value)**
**R-dAI**	**iA**	**healthy (H)**	-0.021, 0.913	0.117, 0.547	0.000, 0.999	-0.066, 0.734
		**depressed (D)**	0.941, **0.005**	0.846, **0.034**	0.697, 0.124	-0.010, 0.985
		**H+D**	-0.116, 0.509	-0.036, 0.837	-0.238, 0.168	-0.295, ***0.085***
**R-vAI**	**iA**	**healthy (H)**	-0.073, 0.713	0.018, 0.927	-0.100, 0.612	-0.159, 0.418
		**depressed (D)**	0.300, 0.564	0.473, 0.343	0.139, 0.793	-0.078, 0.884
		**H+D**	-0.170, 0.336	-0.079, 0.657	-0.323, ***0.062***[†]	-0.354, **0.040**[†]
**R-PI**	**iA**	**healthy (H)**	-0.084, 0.664	0.067, 0.729	0.074, 0.704	0.023, 0.904
		**depressed (D)**	0.877, **0.022**	0.871, **0.024**	0.483, 0.331	0.057, 0.914
		**H+D**	-0.108, 0.536	0.001, 0.994	-0.138, 0.430	-0.181, 0.299
**L-PI**	**iA**	**healthy (H)**	-0.146, 0.451	0.053, 0.787	0.072, 0.712	0.005, 0.979
		**depressed (D)**	0.528, 0.281	0.792, ***0.060***	0.387, 0.449	0.327, 0.527
		**H+D**	-0.187, 0.282	-0.012, 0.947	-0.159, 0.363	-0.178, 0.305
**R-PI**	**eA**	**healthy (H)**	0.093, 0.631	0.140, 0.468	-0.001, 0.996	-0.137, 0.480
		**depressed (D)**	0.452, 0.369	0.873, **0.023**	0.267, 0.609	0.596, 0.212
		**H+D**	0.053, 0.763	0.178, 0.306	-0.106, 0.546	-0.147, 0.400
**R-dAI**	**eA**	**healthy (H)**	-0.086, 0.653	0.052, 0.784	0.021, 0.910	-0.085, 0.656
		**depressed (D)**	0.796, ***0.058***	0.822, **0.045**	0.706, 0.117	0.111, 0.834
		**H+D**	-0.046, 0.788	0.066, 0.701	-0.035, 0.840	-0.180, 0.294

[†] please see also Suppl. figure 2
